# Streptococcus constellatus Brain Abscess in a Middle-Aged Man With an Undiagnosed Patent Foramen Ovale

**DOI:** 10.7759/cureus.34626

**Published:** 2023-02-04

**Authors:** Susan G Wager, Nina K Bourdeau, Joshua D Collins

**Affiliations:** 1 Internal Medicine, Wayne State University School of Medicine, Detroit, USA; 2 Internal Medicine, Henry Ford Health System, Detroit, USA

**Keywords:** ring-enhancing lesions, pfo, tee, cerebral abscess, transesophageal echocardiogram, streptococcus anginus, patent foramen ovale, streptococcus constellatus, brain abscess

## Abstract

Brain abscess is a rare diagnosis. Common sources of infection include direct spread from otic sources, sinuses, or oral cavities, and hematogenous spread from distant sources, including the heart and lungs. Brain abscess with cultures growing oral flora species, in rare cases, may develop from bacteria in the oral cavity entering the bloodstream and then traveling to the brain via a patent foramen ovale. This report highlights a case of brain abscess caused by *Streptococcus constellatus* in a middle-aged man with an undiagnosed patent foramen ovale.

## Introduction

Brain abscess is a rare diagnosis, with a reported incidence of 0.3 to 1.3 per 100,000 people per year [1-3]. In nearly 40% of cases, the source of infection is unknown [4,5]. Infection etiologies are divided into primary sources and secondary sources. Primary sources include the direct introduction of bacteria from penetrating cranial injuries, facial trauma, or brain surgery. Secondary sources include infections with non-neural origins that spread via continuous or hematogenous routes. Continuous spread of infection may seed from otic sources, including otitis and mastoiditis; paranasal, frontal, or ethmoid sinuses; or oral sources, primarily dental infections. Hematogenous spread commonly originates from pulmonary sources, including lung abscesses, empyema, pulmonary arteriovenous malformations, or bronchopulmonary fistula [6]. Cardiac pathologies can also lead to the formation of a brain abscess, including cyanotic congenital heart defects in children, bacterial endocarditis, ventricular aneurysm, and thrombosis [6]. Brain abscess secondary to bacteremia characteristically shows multiple abscesses.

Brain abscesses are typically caused by *Streptococcus* and *Staphylococcus* species, primarily *Streptococcus viridans* and *Staphylococcus aureus* [6]. Rare organisms that may be involved in brain abscess formation include *Streptococcus anginosus* species (*Streptococcus anginosus*, *Streptococcus constellatus*, and *Streptococcus intermedius*). These organisms are a known oral flora recognized for their tendency to form abscesses; however, they are rarely involved in the formation of brain abscesses. This report presents a rare case of brain abscess caused by *Streptococcus constellatus*, in the setting of septicemia in a male patient in his 60s with an undiagnosed patent foramen ovale (PFO).

## Case presentation

A 63-year-old male presented to the emergency department (ED) with intermittent headaches for three months and new-onset fatigue and altered mental status one day after being diagnosed with pneumonia. His presenting vital signs were a temperature of 39.4°C, oxygen saturation of 94% on room air, heart rate of 125 beats per minute, and blood pressure of 119/75 mmHg. Physical examination was positive for tachycardia without murmurs and neurologic findings included left superior quadrantanopia and left upper extremity pronator drift. Initial laboratory evaluation revealed leukocytosis of 25,100 cells/mm^3^ with left shift. Computed tomography (CT) of the brain performed in the ED was suggestive of a subacute infarct in the right inferior parietal lobe with a small subdural hematoma in the superior right parietal lobe. The patient was diagnosed with sepsis secondary to pneumonia and admitted to the hospital for treatment with empiric antibiotics, ceftriaxone, and azithromycin to cover for his known pneumonia.

**Figure 1:**
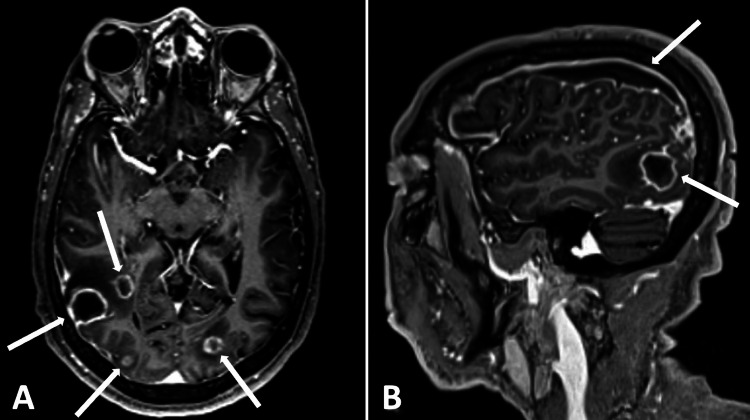
Initial brain MRI demonstrating multifocal ring-enhancing lesions in the parietal and occipital lobes, and right-sided subdural fluid collection. (A) Axial contrast-enhanced 3D MPRAGE; (B) sagittal contrast-enhanced 3D MPRAGE. 3D, three-dimensional; MPRAGE, magnetization-prepared rapid acquisition gradient echo.

Shortly after admission, the patient had two focal tonic-clonic seizures, predominantly affecting the left upper extremity. The patient’s treatment was changed to cover for meningitis with acyclovir, vancomycin, and ampicillin. Electroencephalogram (EEG) showed right frontotemporal central focal impaired seizures. Interictal findings included right temporal intermittent rhythmic delta activity (TIRDA) and right frontal lateralized periodic discharges (LPDs) suggesting an increased risk for focal seizures. Continuous right frontotemporal slowing indicated underlying structural or functional cerebral abnormalities. Lumbar puncture was significant for an elevated white blood cell count of 23 cells/mm^3^ (24% neutrophils) and elevated protein of 102.1 mg/dL with negative gram stain and culture on cerebrospinal fluid. Brain magnetic resonance imaging (MRI) showed multifocal ring-enhancing lesions in the right inferior parietal occipital region with additional hemorrhagic ring-enhancing lesions noted diffusely throughout the brain concerning for hemorrhagic metastases (Figure 1).

Additional workup in the setting of these imaging findings included CT of the chest, abdomen, and pelvis that showed no signs of metastatic or primary malignancy. However, there were partial cavitary opacities in the medial right lower lobe that resembled inflammatory or infectious etiology, consistent with his pneumonia. A transthoracic echocardiogram (TTE) was performed to rule out emboli secondary to infective endocarditis, which showed no evidence of endocarditis. At this time, the neurosurgery team was consulted and proceeded with a craniotomy to obtain a brain biopsy. During the procedure, purulent material was observed, and cultures of the surgical specimen were sent for aerobic, anaerobic, and fungal organisms. Anaerobic cultures grew rare *Streptococcus constellatus*, confirming a diagnosis of brain abscess.

While a diagnosis of brain abscess was confirmed, at this point, the source of the infection was still unknown. Since *Streptococcus constellatus* is commonly found in oral flora and is an organism known to cause abscesses, direct extension from the oral cavity was suspected. The patient was noted to have poor dentition and a cracked right mandibular molar, increasing his risk for contiguous spread from the oral cavity; however, he exhibited no overt signs of dental infection, and a CT of the face showed no evidence of soft tissue infection. Without direct seeding from the oral cavity to the brain, it was suspected that the abscess may have developed from a paradoxical bacterial embolism through a PFO. A transesophageal echocardiography (TEE) bubble study was performed, confirming the presence of a PFO.

The patient was successfully treated with intravenous ceftriaxone and metronidazole during his hospitalization with a plan to complete treatment as an outpatient via a peripherally inserted central catheter (PICC) for a total of four weeks. Post-treatment imaging was not obtained, as the patient was unfortunately lost to follow-up.

## Discussion

Even in the absence of infective endocarditis on echocardiogram, brain abscess should still be considered in the setting of new-onset encephalopathy and undiagnosed brain lesions on imaging with recent or concurrent bacteremia. Cardiac and vascular anomalies such as PFOs and arteriovenous malformations (AVMs) may create an opportunistic environment for the hematogenous spread of oral flora to the brain, resulting in brain abscess formation [5,7-9]. To our knowledge, 19 cases have been reported [7]. Sadahiro et al. highlighted seven patients with reported brain abscesses in the setting of a right-to-left shunt, including PFO (n = 6) and pulmonary arteriovenous shunt (n = 1) identified in the TEE bubble study [7]. Four patients were found to have *Streptococcus intermedius* bacteria, and cultures of three patients grew normal oral flora, all of whom had periodontal disease [7]. Due to the higher sensitivity and specificity of TEE compared to TTE in identifying small PFOs, physicians should perform a TEE bubble study in patients with brain abscesses with oral flora organisms [8,10]. Additionally, in the setting of recurrent brain abscess, closure of PFO or AVM may be considered to decrease the risk for future abscesses. However, further research is needed to better understand the potential benefits and outcomes of PFO closure in these cases [8,11].

Recognized antibiotic treatment for brain abscesses include empiric coverage of *Streptococcus* species and oral anaerobic species. In undifferentiated cases, additional empiric coverage for methicillin-resistant *Staphylococcus aureus* (MRSA) may be considered [12]. A six-to-eight-week antibiotic course is typically recommended for brain abscesses; however, studies have shown shorter courses may be sufficient following surgical intervention, such as in this patient [3,13].

Among the few reported cases of *Streptococcus constellatus* brain abscess, routes of infectious spread included direct seeding from dental infection and hematogenous spread originating from infective endocarditis or paraspinal abscess [14-17]. This patient's case was not only a rare route of infectious spread among cases of brain abscesses but also may be the first reported case of *Streptococcus constellatus* brain abscess caused by hematogenous spread through a PFO. Given the association between brain abscess and oral cavity bacteria, it is important for physicians to educate patients about the value of oral hygiene and improve access to dental care for all patients. These preventative measures may limit the risk of brain abscess formation [18].

## Conclusions

While brain abscesses are uncommon, physicians should consider brain abscesses as a differential diagnosis in patients with neurologic symptoms and suspicious imaging findings. Brain abscesses, in rare cases, may originate from oral flora organisms entering circulation and spreading to the brain through a PFO, even in the absence of endocarditis. Patients with oral flora bacteria isolated from brain abscesses with an unknown source of infection should be assessed for PFO with a TEE bubble study.
